# Association of two single nucleotide polymorphisms rs10407022 and rs3741664 with the risk of primary ovarian insufficiency in a sample of Iraqi women 

**DOI:** 10.22099/mbrc.2020.36371.1477

**Published:** 2020-12

**Authors:** Tamadher Abbas Rafaa, Ahmed AbdulJabbar Suleiman, Mustafa Falah Dawood, Ali Mohammed Al-Rawi

**Affiliations:** 1 University Headquarter, University of Anbar, Ramadi, Iraq; 2 College of Science, University of Anbar, Ramadi, Iraq; 3 College of Education for Pure Science, University of Anbar, Ramadi, Iraq

**Keywords:** Tetra arms PCR, POI, AMH, FSH, LH

## Abstract

Primary ovarian insufficiency (POI) can be a devastating disease impacting women below the age of forty. This involves a major decrease in the amount and quality of oocytes, or ovarian reserve in a woman. The distribution of single-nucleotide polymorphisms, rs10407022 and rs3741664, in Iraqi people and its association with primary ovarian insufficiency is the main objective of this study. The mean of FSH and LH levels of patients with POI was higher than control, while the mean of AMH levels of patients was lower compared to control. For rs10407022, the GT and TT genotypes were positively associated with the risk of POI. For the rs3741664, the AG genotype was negatively associated with the risk of POI. The results lead to the main conclusion that rs10407022 and rs3741664 polymorphisms may significantly affected the serum levels of AMH and FSH and thus affect POI etiology.

## INTRODUCTION

Premature ovarian insufficiency (POI) is a condition previously known as “primary ovarian insufficiency” [1]. POI term is progressively utilized and need being received to envelop diagnostically comparable states, which include untimely ovarian disappointment, hypogonadotropic hypogonadism, and dysgenesis of the ovary [2]. POI is characterized with the accelerated depletion of ovarian reserve including a decrease in the number of a wide variety of residual follicles and deficiency in intercourse hormones, which lead to a reduction in fertility and hypoestrogenism level years before the normal age of menopause [3]. Indeed, for most patients offering with POI, the reason is basically unexplained [4]. Potential POI etiologic factors might be classified as into hereditary, immune system, metabolic brokenness, irresistible, and androgenic factors [2]. 

Anti-Mullerian hormones (AMH), otherwise called Mullerian-restraining substance is a confident marker of ovarian saves. AMH is a dimeric glycoprotein of the changing development of factor-β superfamily, which is associated with development and differentiation. AMH has a potential role in develpoing various ovarian dysfunctions [5]. AMH shows up in serum after birth, increments until pubescence, and logically diminishes in corresponding to ovarian maturing [6]. Indeed, the researchers noted that early ovarian aging and POI are associated with terribly undetectable or low serum AMH levels [7, 8]. Other studies have incontestable that body fluid AMH level could be a higher indicator of ovarian reserve than age alone or alternative indicators like FSH of basal serum, estrogen ,and inhibin B levels [9]. From these findings, AMH levels in serum appear to be the most effective indicator for dysfunction in ovarian reserve [10]. The rs10407022 is a SNP located in 19p13.3. This SNP changes thymine to guanine in the position 146, leading to change amino acid isoleucine to serine in the position 49. The rs3741664 changes G4952A guanine to adnine in the position 4952 [11]. The study was conducted to achieve the goal of investigating the effect of rs10407022 and rs3741664 polymorphisms on the risk of POI.

## MATERIALS AND METHODS

**Subjects:** In this study, forty-five premature ovarian insufficiency women were assessed; patients with POI were recruited from women and children hospital, Ramadi city, Iraq from August 2019 to January 2020, also 45 apparently healthy women from Ramadi city were used as a control group.

**Hormonal assay:** Serum was moderate into (2 days) for entire subjects by ELISA (enzyme-linked immune sorbent assay) technique using FSH, LH ELISA Test Kit (Monobind, United States), the AMH has been estimated using Anti-Mullerian hormone ELISA Kit (Beckman coulter, USA).The age group and hormonal assay were listed in supplementary table. 

**Polymorphism study:** DNA was extracted from blood (peripheral) using a genomic DNA purification mini kit provided by Geneaid (Geneaid Biotech. Ltd, Taiwan). SNPs were identified using accessible National Center for Biotechnology Information (NCBI) database (http://www.ncbi.nlm.nih.gov/SNP). Two special sets of primers were designed specifically for this study. For rs10407022, the sequence of the primers was IF GGACTGGCCTCCAGGTAT, IR CACAGAGGCTCTTGTGAGC, OF ATAGGGGTCTGTCCTGCAC and OR CTCCAGGT GTAGGACCACC. Product size was 279bp for T allele, 244bp for G allele, and 486 bp for outer primers. For rs3741664, the sequence of the primers was IF TTGTGTACCATCCTTTTC TCTCTTCG, IR ACAGGGAGCCCTGGGGACAT, OF CCATCAGTGTGTCTGTCTGCTGG, and OR ACACTGTGGCTCCCTTGACTTCC. The size of products was 191 bp for G allele, 132 bp for A allele and 276 bp for outer primers. 

**Statistical analysis: **Hardy-Weinberg equilibrium was used to evaluate the results., Genotypes and allele frequency of rs10407022 and rs3741664 variation were calculated as percentage frequencies. Differences between controls and patients were evaluated significantly by the calculation of odds ratio (OR) and its 95% CI (confidence interval). 

## RESULTS AND DISCUSSION

The genotypic and allelic distributions of POI and control groups were show in Table 1. The frequency of the observed genotypic polymorphisms in control did not show significant deviation from the expected values based on the Hardy-Weinberg equilibrium (P>0.05). For rs10407022, the GT and TT genotypes were significantly associated (P=0.001) with elevation risk of POI (Table 1). The AG and GG genotypes of the rs3741664, decreased the risk of POI. For SNP rs10407022, it has been observed that TT genotypes had higher AMH levels compared to GT and GG. 

**Table 1:** The genotypes of the rs10407022 and rs3741664 polymorphisms in POI and control groups

**Polymorphisms**	**Controls**	**Patients**	**OR**	**95% CI**	**P**
**rs10407022**					
**GG**	28	12	1.0	-	-
**GT**	11	22	4.66	1.73-12.5	0.002
**TT**	6	11	4.27	1.28-14.2	0.018
					
**rs3741664**					
**AA**	21	33	1.0	-	-
**AG**	20	11	0.35	0.14-0.87	0.025
**GG**	4	1	0.15	0.02-1.52	0.111

For rs10407022, it has been observed that TT genotypes had higher AMH levels compared to GT and GG. Genotypes are shown in Table 2. FSH and LH serum levels were not positively correlated. For SNP rs3741664, the genotypes AA had elevated FSH levels compared to AG and GG genotypes. LH and AMH levels were not associated positively.

**Table 2:** Association of the AMH SNPs with hormonal value

**rs10407022**				
**Variables**	**GG**	**GT**	**TT**	**P**
**AMH**	0.40±1.25	1.37±1.21	3.44±5.25	0.03*
**FSH**	61.24±16.65	44.58±24.92	51.64±54.65	0.12
**LH**	53.21±16.25	62.54±26.21	66.11±42.25	0.09
**rs3741664**				
**Variables**	**AA**	**AG**	**GG**	**P**
**AMH**	0.67±0.69	0.59±0.15	0.86±1.12	0.16
**FSH**	78..33±19.78	65.58±24.92	62.13±22.35	0.02*
**LH**	64.36±14.45	68.44±34.16	67.24±48.16	0.11

Several studies prove that AMH plays an inhibitory function in the follicles recruitment. Polymorphisms in the AMH gene could affect the biological activities of the hormone, which play an essential role in differentiation, maturation, and follicles development [12]. Kevenaar et al., [13] recommended that genotype GG of AMHR2 may end up with diminished AMH sign.Due to the nature of its location in the promoter region, it can cause disequilibrium with different SNPs. The AMH signaling diminish could lead to magnified primordial cyst recruitment [14]. *AMH* and *AMHR2* variations couldcontribute to the change in the threshold of ovary FSH [14]. These polymorphisms seems to affect biological activities of the hormone, subsequently influencing follicle enrollment and advancement [12].

The G146T/rs10407022 polymorphism which are allocated in the upstream region of the *AMH* geneis responsible for the stability and folding of a protein. Thus SNPs in this region could affect AMH bioactivity. This change in the AMH gene can produce inactive protein.[11, 12]. The polymorphism of rs10407022 affect AMH bioactivity, but not it’s processing. That is the reason why AMH and AMHR2 polymorphisms can anticipate the embryos number production and seem to have reduced signaling/function of minor allele carriers. [15]. The SNP is related to higher follicular phase estradiol levels in normal-ovulatory women and thereby supporting the thought of AMH regulating FSH-sensitivity in the ovary [13]. Genotype TT of the rs10407022 diminishes the affect-ability of antral follicles to FSH less than the GG genotype. The women with TT genotype were portrayed by lower protein bioactivity than individuals with the GG genotype [12].

SNPs rs3741664 in the AMHR gene was associated, in a positive manner, with the level of AMH, and FSH in serum [16]. The Ser/Ser variant (TT genotype) has a negligible effect in minimizing the FSH sensitivity of antral follicles. *In vitro* studies have reported that protein with Ser/Ser residue has apparent reduced bioactivity compared to the Ile/Ile protein, and thus, the T/Ser allele had follicle with lower total number compared with the other groups [11].

## Supplementary Materials

Supplement
