# Supplementary material for: Association of two single nucleotide polymorphisms rs10407022 and rs3741664 with the risk of primary ovarian insufficiency in a sample of Iraqi women
Source: Mol Biol Res Commun. 2020 Dec;9(4):141–4. doi: 10.22099/mbrc.2020.36371.1477 (PMC7731970; doi:10.22099/mbrc.2020.36371.1477)
Supplement: Supplement [file mbrc-9-141-s001.pdf]

Table S2.Clinical characteristics of females experiencing POI compared to the control group.

Characteristic	POI(45)	Control (45)	P-Value
Age(year)	28.54±4.25	27.65±3.86	0.872 NS
FSH(mlU\ml)	68.24±23.24	13.12±11.75	0.009 Highly Sign
LH(mlU\ml)	66.58±57.45	16.22±21.42	0.007 Highly sign
AMH(mlU\ml)	0.72±0.44	6.12±8.24	0.011 sign

Values are: mean ± SD. P-Values are calculated using the ANOVA test. NS = non-significant
